# Oseltamivir Resistance in Human Influenza A(H5N1) and A(H7N9) Infections: A Mini Review

**DOI:** 10.1093/ofid/ofag393

**Published:** 2026-07-01

**Authors:** Yacine Abed, Guy Boivin

**Affiliations:** Research Center in Infectious Diseases of the Centre Hospitalo-Universitaire de Québec (CHUQ), Pavillon CHUL and Laval University, Québec City, Québec, Canada; Research Center in Infectious Diseases of the Centre Hospitalo-Universitaire de Québec (CHUQ), Pavillon CHUL and Laval University, Québec City, Québec, Canada

**Keywords:** H5N1, H7N9, influenza, oseltamivir, resistance, treatment

## Abstract

Avian influenza viruses (AIVs) have been reported to cause infections in humans following avian-to-human transmission, resulting in a range of clinical outcomes. A(H5N1) and A(H7N9) infections, which constitute the majority of human AIV cases, are responsible for severe infections leading to high mortality. The neuraminidase inhibitor oseltamivir is expected to play a major role for the control of AIV infections in humans. However, the emergence of resistance may compromise the impact of antiviral therapy. The objective of this article is to review human cases of A(H5N1) and A(H7N9) infections for which mutations of oseltamivir resistance were detected. Neuraminidase mutations rapidly occurred in a subtype-specific manner, with H274Y and N294S substitutions predominating in A(H5N1) cases and the R292K substitution in A(H7N9) cases. Serious clinical outcomes and mortality were seen in most A(H5N1) and A(H7N9) cases despite oseltamivir therapy, thus highlighting the need for improving antiviral strategies against these AIVs.

Influenza A viruses are highly transmissible pathogens responsible for annual epidemics and occasional but devastating pandemics. These viruses contain 2 surface glycoproteins, the hemagglutinin (HA) and neuraminidase (NA), whose genetic differences allow their classification into 16 HA and 9 NA avian subtypes [1]. The wild aquatic birds are the main natural reservoir of influenza A viruses [2], including waterfowl within the Anseriformes order (ducks, geese, and swans) and Charadriiformes order (gulls, terns, and shorebirds) [3]. Avian influenza A viruses (AIVs) generally cause few signs of disease in infected wild birds, with viral replication mainly occurring in the gastrointestinal tract, resulting in fecal shedding [4]. Therefore, fecal-oral transmission, involving contaminated water is considered the main route of AIV transmission among wild birds [5]. AIV may also transit from wild waterfowl into terrestrial domestic chickens and turkeys, mainly via a direct contact route [6]. Based on the clinical outcome of infection in chickens, AIV can be classified into 2 groups: low pathogenic AIV (LPAIV), consisting of viruses that cause either no signs of disease or mild clinical manifestations, and high pathogenic AIV (HPAIV), including viruses that induce severe hemorrhagic disease leading to high mortality rates [7]. Therefore, HPAIVs constitute a significant threat to animal welfare as well as to the poultry industry.

The LPAIV-to-HPAIV transition could be attributed to a molecular mechanism consisting of the acquisition of nucleotides coding for basic amino acids within the proteolytic cleavage site of HA [5]. The resulting multibasic cleavage site allows the activation of the HA cleavage by ubiquitous proteases, which results in systemic dissemination in terrestrial poultry [8]. Such events have been described in AIV harboring an HA protein of H5 and H7 subtypes [9]. HPAIV infections that occur in poultry can spill back into wild birds, which results in further dissemination of the virus through birds’ migratory flows.

LPAIV and HPAIV have been reported to cause infections in humans following avian-to-human transmission [10]. AIVs of different HA subtypes have been associated with human infections, but the most frequently reported human cases have involved AIVs possessing an HA of H5, H7, and H9 subtypes [11]. Noteworthy, the severity of illness in humans does not correlate with the pathogenicity of AIV in poultry, as LPAIVs and HPAIVs can cause mild to severe illnesses in infected humans [12, 13]. The majority of human cases of AIV infections have involved A(H5N1) and A(H7N9) viruses. Moreover, an increasing concern has been raised with regard to the potential for these variants to start a new influenza pandemic [14, 15]. So far, no sustained human-to-human transmission has been reported for AIV.

Influenza NA can be separated into 2 groups: group 1, which includes N1, N4, N5, and N8, and group 2, which includes N2, N3, N6, N7, and N9. The NA inhibitors (NAIs) available for the control of seasonal influenza A infections, mostly caused by A(H1N1) and A(H3N2) strains, represent an important option for the control of human cases of AIV infections. This antiviral class includes oseltamivir, zanamivir, peramivir, and laninamivir, which target the influenza NA protein, whose NA activity is responsible for virion release from infected cells and virus mobility in mucus [16]. The most widely prescribed NAI (ie, oseltamivir) has been recommended as a first-line antiviral agent against A(H5N1) and A(H7N9) infections [17, 18]. Stockpiling of oseltamivir for use in the advent of a new influenza pandemic has been widely adopted [19].

As observed for other antimicrobial agents, drug-resistant influenza variants may emerge under NAI pressure [20]. Such events have been reported in numerous clinical reports in the context of seasonal influenza A(H1N1), A(H1N1)pdm09, A(H3N2), and B infections [20–22]. NA substitutions that affect susceptibility may occur at catalytic residues (292; N2 numbering, here and throughout the text) or framework residues (119, 222, 274, 294). Herein, we review human cases of oseltamivir resistance associated with A(H7N9) and A(H5N1) infections. We searched publications in English on Medline, Embase, and Google Scholar up to 15 March 2026 using the terms “A(H5N1) human infections” and “A(H7N9) human infections,” as well as avian influenza infections combined to the terms “antiviral resistance” and “oseltamivir resistance.”

## HIGHLY PATHOGENIC INFLUENZA A(H5N1) HUMAN INFECTIONS

The HPAI A(H5N1) viruses were discovered in domestic waterfowl in southern China in 1996 and caused large poultry outbreaks in Hong Kong in 1997 [23]. These viruses were subsequently associated with human infections in Asia and were characterized by a high mortality rate [24]. Despite the efficient control of the 1997 A(H5N1) outbreak, HPAIV A(H5N1) viruses were not eradicated in birds. They reemerged in 2003 to become widespread in different regions of the world, including Asia, Africa, Europe, and the Middle East, causing poultry outbreaks and sporadic human infections [25]. Since 2003, >23 countries have reported >890 sporadic human infections with A(H5N1) viruses to the World Health Organization.

## OSELTAMIVIR RESISTANCE IN HIGHLY PATHOGENIC A(H5N1) HUMAN INFECTIONS

The oseltamivir-resistant A(H5N1) clinical cases identified in this review (Table 1) occurred during the 2005–2006 period [26–28]. No other cases have been reported to the World Health Organization after 2006 [29]. These cases involved A(H5N1) strains from the A/goose/Guangdong/1/96 lineage.

**Table 1.** Human Cases Associated With Oseltamivir-Resistant Influenza A(H5N1) Viruses

				Fold Increase in Antiviral IC50 (Phenotype) ^a^				
A(H5N1) Isolate ^b^	Sex, Age	NA Substitution in N2 Numbering	Antiviral/Treatment or Prophylaxis, Duration	Osel	Zan	Per	Clinical Outcome	Reference
A/Hanoi/30408/2005	F, 14 y	H274Y	Osel/prophylaxis, 3 d	1766 (HRI)	1 (NI)	…	Discharged	[28]
		N294S		20.8 (RI)	3.5 (NI)	…		
A/Vietnam/CL2009/2005	F, 13 y	H274Y	Osel/treatment, 4 d	…	…	…	Death	[26]
A/Vietnam/CLX/2005	F, 18 y	H274Y	Osel/treatment, 9 d	…	…	…	Death	[26]
A/Egypt/14724/2006	F, 16 y	N294S	Before osel and after treatment for 2 d	93 (RI)	3 (NI)	4 (NI)	Death	[27]
A/Egypt/14725/2006	M, 26 y	N294S	Before osel and after treatment for 2 d	…	…	…	Death	[27]

Abbreviations: F, female; IC50, half-maximal inhibitory concentration; M, male; NA, neuraminidase; Osel, oseltamivir; Per, peramivir; Zan, zanamivir.

^a^HRI (highly reduced inhibition), >100-fold increase vs wild type as determined by NA inhibition assays; NI (normal inhibition), <10-fold increase vs wild type; RI (reduced inhibition), between 10- and 100-fold increase vs wild type. Ellipses (…) indicate *not determined.*

^b^Pathogenicity for each isolate: highly pathogenic avian influenza virus.

The first case of oseltamivir resistance in an A(H5N1) virus (A/Hanoi/30408/2005) involved a 14-year-old Vietnamese girl. During 1 week (14–21 February 2005), the child had cared for her 21-year-old brother with a documented A(H5N1) infection. She developed a mild cough and fever on 22 February and received a prophylactic dose of oseltamivir (75 mg once a day) during 3 days (24–27 February). Viral clones prepared with the isolate obtained on 27 February revealed the presence of oseltamivir-resistant A(H5N1) variants harboring the H274Y or N294S NA substitutions. She was admitted to a hospital in Hanoi for observation and given a therapeutic dose of oseltamivir (75 mg twice daily for 7 days starting on 28 February). At the end of this treatment, no virus was isolated from her specimens. She recovered and was discharged from the hospital on 14 March [28].

The following case (A/Vietnam/CL2009/2005) involved a 13-year-old Vietnamese girl admitted to a hospital on 22 January 2005 with influenza-like illness. The day before, her mother (aged 35 years) had died of influenza A (H5N1) virus infection. The child received an initial 75-mg dose of oseltamivir on admission, followed by 2 additional 75-mg doses within the first 24 hours after admission. Treatment was then continued for 4 days. On 25 January (the fourth day of oseltamivir treatment), the child's respiratory condition worsened, and supplemental high-dose oxygen was given with no improvement of her respiratory condition. She was then intubated, with mechanical ventilation begun on 27 January, but she died on 28 January. The influenza A (H5N1) virus isolated from a throat swab obtained on the fourth day of oseltamivir treatment (25 January 2005) contained the H274Y NA substitution [26].

In the same clinical report, another case (A/Vietnam/CLX/2005) involved a 18-year-old female who was admitted to the hospital on 5 January 2005 with pneumonia. On admission, oseltamivir treatment was started with antibiotics. Supplemental oxygen was given during the first 3 days of admission, but on the fourth day after admission, she was intubated and underwent ventilation because of progressive respiratory failure. On the ninth day after admission, an oseltamivir-resistant A(H5N1) variant (H274Y) was isolated from her throat. Then, her temperature rose and leucocyte count increased to 18 000 cells/mm^3^ with 90% neutrophils. Initial antibiotics were replaced, but her respiratory and clinical conditions continued to deteriorate. She died of respiratory failure on 19 January 2005 [26]. Of note, the NA sequences from swabs obtained at admission and after 2 days of treatment showed a pure wild type (H274) virus genotype [26].

The N294S NA substitution was detected in 2 Egyptian cases with A(H5N1) infections from clade 2.2 that occurred in December 2006 in the Nile Delta region of Lower Egypt [27]. The cases A/Egypt/14724/2006 and A/Egypt/14725/2006 involved a 16-year-old female and a 26-year-old male who participated in the slaughter of household ducks on December 13 and developed influenza-like illness between 15 and 19 December. A throat swab was collected from each case before beginning oseltamivir treatment on 21 December according to World Health Organization guidelines [27]. A second swab was collected after 2 days of treatment. The 2 patients died from pneumonia complicated by adult respiratory distress syndrome. Sequence analyses of the samples revealed the presence of the N294S NA substitution, which was present before starting oseltamivir therapy [27].

## INFLUENZA A(H7N9) HUMAN INFECTIONS

The first human case of infection with influenza A(H7N9) virus was reported in China in March 2013 [12]. Although not pathogenic in poultry, this virus was responsible for 4 successive annual epidemic waves (from 2013–2014 to 2016–2017) that caused 1567 human infections resulting in 615 deaths [30]. The circulation of A(H7N9) viruses has gradually disappeared, and human infections with the A(H7N9) virus have been eliminated in China with poultry vaccination [31]. During the fifth wave, which occurred from October 2017 to July 2018, the isolates were found to contain multiple basic amino acid insertions in the cleavage site of the HA, indicating that the virus had acquired the HPAIV phenotype [32].

Most human cases of LPAIV and HPAIV A(H7N9) infections involved people with occupational exposure, including farmers with direct contact with poultry, workers involved in the culling, veterinarians, as well as visitors of live bird markets [33]. The severity of the disease did not seem to vary significantly between HPAIV and LPAIV infections [34]. Pneumonia and respiratory failure were described in the majority of human H7N9 cases reported in China, and the infection resulted in high rates of hospitalization, admission to intensive care units, and death [33, 34].

## OSELTAMIVIR RESISTANCE IN LOW PATHOGENIC INFLUENZA A(H7N9) HUMAN INFECTIONS

The A/Taiwan/1/2013 case involved a 53-year-old male patient who was admitted on 16 April 2013 because of fever for 3 days with a suspected A(H7N9) infection after returning from Suchow, Jiangsu province, China, on 9 April 2013. Two throat-swab specimens tested negative by reverse transcription polymerase chain reaction (RT-PCR) for A(H7N9) on 17 and 20 April. Oseltamivir treatment (75 mg twice daily) was started on 16 April. A follow-up chest radiograph on 18 April revealed interstitial pneumonia with progressive dyspnea on 19 April. The dose of oseltamivir was subsequently increased to 150 mg twice daily. Endotracheal intubation and mechanical ventilator support were given because of respiratory failure. He was administered extracorporeal membrane oxygenation on 22 April. Very high A(H7N9) viral loads were found in sputum and throat swab specimens collected on 20 and 22 April [35]. The viruses in clinical samples collected after 6 days of oseltamivir therapy were grown in chicken eggs before plaque purifications. This procedure resulted in the identification of different NA substitutions associated with reduced susceptibility to NAIs, such as R292K, I222R/K, and E119V [36].

The A/Shanghai/1/2013 case involved a 87-year-old man with chronic obstructive pulmonary disease and hypertension who was hospitalized on 15 February 2013, with severe bilateral pneumonia, leukopenia, and lymphocytopenia. Oseltamivir treatment was started on 25 February. Clinical samples from the patient were positive for A(H7N9) when tested by real-time RT-PCR on 26 February. The patient’s clinical condition deteriorated, and he declined admission to the intensive care unit and intubation. He died from refractory hypoxemia 13 days after the onset of illness. The sample throat swab specimen obtained after 2 doses of oseltamivir treatment revealed that the influenza A/Shanghai/1/2013 (H7N9) virus contained the R292K substitution as a mixture with the wild type virus [12, 37].

Two cases, A/Shanghai/4821T/2013 and A/Shanghai/5083T/2013, were part of a series of 14 Chinese patients admitted to the Shanghai Public Health Clinical Center between 4 and 20 April 2013 for A(H7N9) infection [38]. The 2 patients developed pneumonia and further deteriorated to become dependent on extracorporeal membrane oxygenation. The first case (A/Shanghai/4821T/2013) was a 88-year-old man with comorbidities (hypertension, diabetes, and chronic obstructive pulmonary disease) who first received oseltamivir treatment (150 mg twice daily) for 4 days but then switched to peramivir (0.6 g once daily) for the subsequent 6 days. He died after 12 days of hospitalization. The second case (A/Shanghai/5083T/2013) was a 56-year old man who had no comorbidities. He received different doses of oseltamivir (75 mg twice daily, 150 mg once daily, and 75 mg once daily) during 13 days starting from the day of admission before a switch to peramivir (0.6 g once daily) for the subsequent 4 days. The R292K NA mutation was detected in the clinical sample obtained after 4 and 9 days of oseltamivir treatment from the 88- and 56-year-old men, respectively.

## OSELTAMIVIR RESISTANCE IN HIGHLY PATHOGENIC INFLUENZA A(H7N9) HUMAN INFECTIONS

The A/Guangdong/17SF006/2017 case involved a 56-year-old man with diabetes and hypertension who lived in Guangdong province, China. He raised chickens and noticed that some of them were sick and dying weeks before his illness. On 7 January 2017 (day 1 of illness), he had a fever and cough and, shortly thereafter, shortness of breath. On 10 January (day 4), he was admitted to a local hospital where bilateral pneumonia was diagnosed. Due to the history of contact with poultry, oseltamivir treatment (75 mg twice daily) was started. Two days later, his clinical condition deteriorated markedly, with progression to severe respiratory failure, hypoxia, and coma. The presence of A(H7N9) virus in an endotracheal aspirate was confirmed by RT-PCR, and sequencing of the NA revealed the presence of R292K substitution as a mixed population. He was transferred to the intensive care unit for invasive mechanical ventilation. He died 39 days later after contracting various microbial infections (cytomegalovirus, *Pseudomonas aeruginosa*, *Acinetobacter baumannii*) [39, 40].

The A/Guangdong/17SF003/2016 case involved a 43-year-old female with normal medical conditions who developed high fever, chills, headache, mild cough, and shortness of breath. She sought medical care 2 days after illness onset and received antibiotics, antipyretics, and oseltamivir (75 mg twice daily), but her symptoms did not improve. On day 3, she was transferred to another hospital, where the oseltamivir dose was increased (150 mg twice a day from day 3 until day 21) with introduction of peramivir on day 7 (600 mg twice daily until day 21). On day 9, lung damage was observed with rapid progression of opacities and tissue consolidation. On day 13, a notable decrease in opacities and tissue consolidation was observed in the lower lobes of both lungs. She recovered and was discharged on day 22. A respiratory sample on day 3 was positive for A(H7N9) by RT-PCR, and the NA protein of the isolate contained the R292K substitution as a mixed population with the wild type virus [13].

The following 5 oseltamivir-resistant A(H7N9) variants are part of a study on 30 HPAI A(H7N9) human cases that occurred between December 2016 and April 2019 in the mainland of China [40]. Minimal clinical information was provided in this study. The first 3 cases involved the catalytic R292K substitution: the first was identified as a pure population (100% K) for the fatal case, A/Shaanxi/32287/2017, with no information available regarding oseltamivir status; the second and third were identified as mixed populations of R292K/R for the A/Guangdong/17SF032/2017 and A/Guangdong/17SF064/2017 cases. The last 2 cases harbored an E119V (A/Guangdong/17SF037/2017) or H274Y (A/Guangxi/18908/2017) framework substitution [40].

## CONCLUDING REMARKS

NAI and oseltamivir in particular are recommended for the treatment of patients with A(H7N9) and A(H5N1) influenza infections. It is also recommended that NAI treatment be started as early as possible, preferably within 48 hours of disease onset, to ensure the best clinical benefits. Such recommendation is primarily based on clinical studies involving seasonal influenza viruses [41]. In our reviewed cases, high fatality rates were observed in patients infected with A(H5N1) even when oseltamivir therapy was started within 2 days after the onset of illness, as for the 14- and 13-year-old cases (Table 1). Accordingly, in a randomized controlled trial of standard-dose oseltamivir including 17 hospitalized patients (13 adults and 4 children) with A(H5N1) infection, 15 patients died [42]. However, in larger observational studies reporting antiviral effectiveness in patients infected with A(H5N1), survival benefit was significantly higher in oseltamivir-treated individuals as compared with untreated patients [43]. This was particularly true when oseltamivir was started within 2 days of symptom onset [44, 45]. Poor clinical outcomes were similarly observed for the reviewed HPAIV and LPAIV A(H7N9) cases, including severe pneumonia/extracorporeal membrane oxygenation and death. Of note, there was a long delay in starting oseltamivir treatment for some cases, up to 10 days for the 87-year-old man infected with A/Shanghai/1/2013 (Table 2). This is in accordance with previous observations where inconsistent conclusions were reached regarding the impact of NAI on the outcomes of patients with H7N9 infection [10, 34]. Here again, observational studies involving a larger number of cases reported that initiation of oseltamivir treatment within 2 days from symptom onset was associated with important clinical benefits, such as a lower risk of death, a shorter hospital stay, and a lower risk of acute respiratory distress syndrome and mechanical ventilation. Yet, delayed initiation of treatment >5 days significantly increased the risk of death [46].

**Table 2.** Human Cases Associated With Oseltamivir-resistant Influenza A(H7N9) Viruses

				Fold Increase in Antiviral IC50 (Phenotype) ^a^				
A(H7N9 Isolate: Pathogenicity	Sex, Age	NA Substitution in N2 Numbering	Antiviral/Tx or Px, Duration	Osel	Zan	Per	Clinical Outcome	Reference
A/Taiwan/1/2013: LPAIV	M/53 y	E119V	Osel/tx, 6 d	84 (RI)	9 (NI)	1 (NI)	Severe pneumonia, ECMO; outcome?	[35, 36]
		I222K		32 (RI)	8 (NI)	6 (NI)		
		I222R		37 (RI)	12 (RI)	12 (RI)		
		R292K		>10 000 (HRI)	55 (RI)	1587 (HRI)		
A/Shanghai/1/2013: LPAIV	M/87 y	R292K/R	Osel/tx, 1 d	714 (HRI)	…	…	Death	[12, 37]
A/Shanghai/4821T/2013: LPAIV	M/88 y	R292K	Osel/tx, 4 d	…	…	…	Death	[38]
A/Shanghai/5083T/2013: LPAIV	M/56 y	R292K	Osel/tx, 9 d	…	…	…	Severe pneumonia, ECMO; outcome?	[38]
A/Guangdong/17SF006/2017: HPAIV	M/56 y	R292K/R	Osel/tx, 2 d	0.38 (NI)	0.98 (NI)	…	Death	[39, 40]
A/Guangdong/17SF003/2016: HPAIV	F/43 y	R292K/R	Osel/tx, 2 d	0.66 (NI)	0.94 (NI)	…	Discharged	[13, 40]
A/Guangdong/17SF032/2017: HPAIV	F/45 y	R292K/R	Osel/tx, ?	92.03 (RI)	4.18 (NI)	…	Outcome?	[40]
A/Guangdong/17SF064/2017: HPAIV	M/55 y	R292K/R	Osel/tx, ?	3.83 (NI)	4.73 (NI)	…	Death	[40]
A/Shaanxi/32287/2017: HPAIV	M/67 y	R292K	?	>1000 (HRI)	26.83 (RI)	NT	Death	[40]
A/Guangdong/17SF037/2017: HPAIV	M/66 y	E119V	Osel/tx, ?	55.43 (RI)	2.21 (NI)	NT	Discharged	[40]
A/Guangxi/18908/2017: HPAIV	M/53 y	H274Y	Osel/tx, ?	21.89 (RI)	1.13 (NI)	NT	Death	[40]

Abbreviations: ECMO, extracorporeal membrane oxygenation; F, female; HPAIV, highly pathogenic avian influenza virus; IC50, half-maximal inhibitory concentration; LPAIV, low pathogenic avian influenza virus; M, male; NA, neuraminidase; Osel, oseltamivir; Per, peramivir; px, prophylaxis; tx, treatment; Zan, zanamivir.

^a^HRI (highly reduced inhibition), >100-fold increase vs wild type as determined by NA inhibition assays; NI (normal inhibition), <10-fold increase vs wild type; RI (reduced inhibition), between 10- and 100-fold increase vs wild type. Ellipses (…) indicate *not determined.*

Our review revealed the rapid emergence of NA mutations responsible for reduced susceptibility to NAI—in some cases, after only 1 day (A/Shanghai/1/2013) or 2 days (A/Guangdong/17SF003/2016, A/Guangdong/17SF006/2017) of oseltamivir treatment (Table 2). Interestingly, the NA substitution conferring resistance to NAI occurred generally in a subtype-specific manner. The framework H274Y substitution is the primary marker of resistance to oseltamivir and peramivir in influenza viruses of the N1 subtype, such as A(H1N1) and A(H5N1). Other framework substitutions include the N294S variant (oseltamivir resistance) and E119V or E119D variant (cross-resistance to different NAIs) [20]. By contrast, the catalytic R292K substitution seems to be specific to the N2 and N9 subtypes, such as A(H3N2) and A(H7N9) viruses. Accordingly, the oseltamivir-resistant A(H5N1) variants reviewed here contained H274Y and/or N294S substitutions (Table 1), whereas the R292K substitution predominated among oseltamivir-treated HPAIV and LPAIV A(H7N9) variants (Table 2). Surveillance studies in birds have also detected A(H5N1) variants with H274Y or N294S substitutions and A(H7N9) variants with the R292K substitution [29, 47]. Of importance, the phenotype of reduced inhibition could not be confirmed by NA inhibition assays in some cases. This could be explained by the substantially reduced activity of the NA R292K variant as previously suggested [37] and to a low proportion of the K residue in the R292K/R mixed populations, where K accounted for <10% in some cases [40]. By contrast, a sharp increase in oseltamivir half-maximal inhibitory concentration ratio (>1000-fold) could be seen in mutants with a pure R292K population, as is the case for the A/Shaanxi/32287/2017 H7N9 variant (Table 2).

The link between oseltamivir treatment/prophylaxis and emergence of the drug-resistant NA mutation could be made in a few A(H5N1) and A(H7N9) cases for which pretherapy viral NA sequences were available (eg, A/Vietnam/CLX/2005, Table 1; A/Guangdong/17SF003/2016, Table 2). Nevertheless, the presence of the N294S substitution before oseltamivir treatment in the 2 Egyptian A(H5N1) cases and the absence of sequence information in pretherapy samples from many cases suggest possible transmission of resistant viruses. Prospective surveillance studies of human cases with AIV infections should include a search for NAI resistance genotypic markers independent of NAI use.
